# Supplementary material for: Genetic Relatedness of 5-Year Isolates of Clostridioides difficile Polymerase Chain Reaction Ribotype 017 Strains in a Hospital
Source: Antibiotics (Basel). 2021 Oct 9;10(10):1229. doi: 10.3390/antibiotics10101229 (PMC8532766; doi:10.3390/antibiotics10101229)
Supplement: Supplementary file 1 [file antibiotics-10-01229-s001.zip › antibiotics-1388203-supplementary.pdf]

Supplementary Table S1. Common multilocus variable-number tandem repeat analysis (MLVA) types of *Clostridioides difficile* by year

No. of isolates / MLVA type (clonal complex)	Year					Total
	2009	2010	2011	2012	2013	
5 (CC-A)	0	3	3	0	0	6
17 (CC-A)	0	0	0	0	8	8
77 (CC-A)	0	4	1	0	0	5
78 (CC-A)	0	1	0	17	15	33
80 (CC-A)	0	1	0	0	4	5
82 (CC-A)	0	2	5	0	0	7
83 (CC-A)	0	11	10	0	0	21
111 (CC-A)	1	8	0	0	0	9
112 (CC-B)	5	1	0	0	0	6
123 (CC-A)	0	5	0	0	0	5
124 (CC-A)	0	5	13	0	0	18
125 (CC-D)	0	0	0	2	6	8
Subtotal no. of isolates	6	41	32	19	33	131
Total no. of MLVA types	16	22	19	11	12	64
Total no. of isolates	20	59	50	29	42	200

Supplementary Table S2. Comparison of antimicrobial resistance among isolates from CC-A and the other CCs

		cluster A (N=163)	others (N=37)	*p* value	rho, *p* value
clindamycin	Resistance rate, N(%)	163 (100)	37 (100)		
	MIC, median (1Q, 3Q)	512 (512, 512)	512 (512, 512)	0.105**	0.115, 0.105
moxifloxacin	Resistance rate, N(%)	162 (99.4)	36 (97.3)	0.337*	
	MIC, median (1Q, 3Q)	64 (64, 64)	64 (32, 64)	<0.001**	0.46, <0.001
vancomycin	Resistance rate, N(%)	1 (0.6)	1 (2.7)	0.337*	
	MIC, median (1Q, 3Q)	0.38 (0.06, 0.5)	0.19 (0.02, 0.38)	0.011**	0.18, 0.011
piperacillin/tazobactam	Resistance rate, N(%)	0 (0)	0 (0)		
	MIC, median (1Q, 3Q)	16 (16, 16)	16 (8, 16)	0.03**	0.154, 0.03
rifaximin	Resistance rate, N(%)	145 (89)	32 (86.5)	0.775*	
	MIC, median (1Q, 3Q)	128 (64, 128)	128 (128, 128)	0.599**	-0.037, 0.6
metronidazole	Resistance rate, N(%)	0 (0)	0 (0)		
	MIC, median (1Q, 3Q)	0.25 (0.13, 0.25)	0.25 (0.13, 0.25)	0.238**	-0.084, 0.239

*, p for chi-square test

**, p for M-W U test

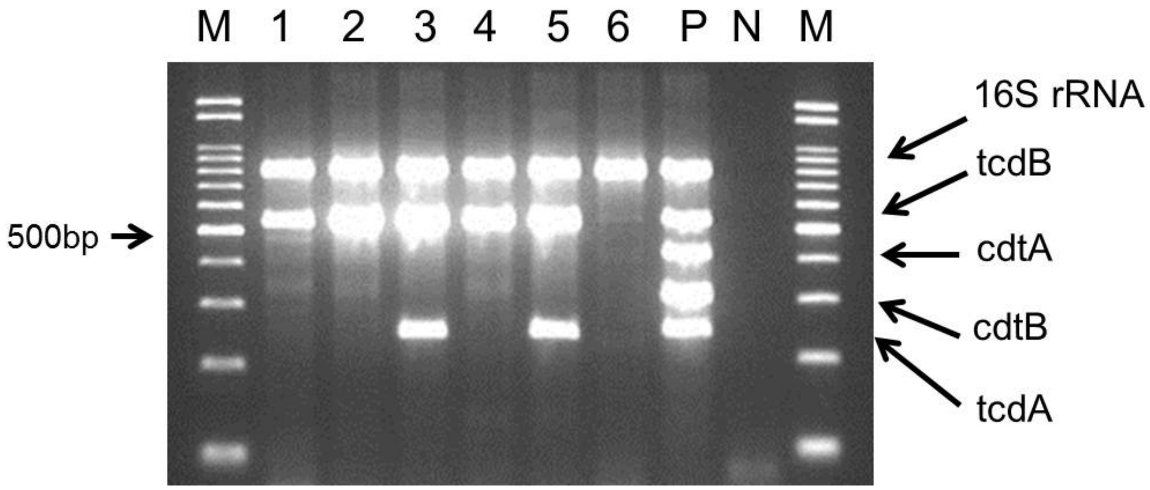

Supplementary Figure S1. Agarose gel electrophoresis of multiplex PCR for toxin genes of *Clostridioides difficile*. Lane 1, 2 and 4, tcdA negative, tcdB postive strains (A-B+CDT-) ; Lane 3 and 5, tcdA/B positive strains (A+B+CDT-) ; Lane 6, non-toxigenic strain (A-B-CDT-). P, positive control (BI/NAP1/027 strains) ; N, negative control ; M, 100bp DNA marker
